# Pooled safety analyses of ALK-TKI inhibitor in ALK-positive NSCLC

**DOI:** 10.1186/s12885-017-3405-3

**Published:** 2017-06-12

**Authors:** Qian Zhu, Hao Hu, De-Sheng Weng, Xiao-Fei Zhang, Chang-Long Chen, Zi-Qi Zhou, Yan Tang, Jian-Chuan Xia

**Affiliations:** 1 State Key Laboratory of Oncology in Southern China, Collaborative Innovation Center for Cancer Medicine, Sun Yat-sen University Cancer Center, Guangzhou, 510060 People’s Republic of China; 2 Department of Thoracic Surgery, Medical College of Nanchang University, Nanchang, 330006 People’s Republic of China; 3 Department of Biotherapy, Sun Yat-Sen University Cancer Center, Guangzhou, 510060 People’s Republic of China

**Keywords:** Crizotinib, Ceritinib, Alectinib, Anaplastic lymphoma kinase, Tyrosine kinase inhibitors, Non-small-cell lung cancer

## Abstract

**Background:**

The anaplastic lymphoma kinase tyrosine kinase inhibitors (ALK-TKIs) have been administered to patients with ALK-positive non-small cell lung cancer for a long period of time and show a promising response. However, the differences in the toxicity profiles among these drugs are still unclear.

**Methods:**

We performed a comprehensive search of the MEDLINE, EMBASE, WEB OF SCIENCE and COCHRANE databases from the drugs’ inception to May 2016 to identify clinical trials. Severe adverse events (AEs) (grade ≥ 3) based on the ALK-TKI type were analysed.

**Results:**

Seventeen trials published between 2011 and 2016, including a total of 1826 patients, were eligible for analysis. Patients in 10 trials (*n* = 1000) received crizotinib, patients in 5 trials (*n* = 601) received ceritinib and patients in 2 trials (*n* = 225) received alectinib. The overall frequencies of treatment-related death and AEs due to treatment withdrawal were 0.9% (12/1365) and 5.5% (85/1543), respectively. Moreover, the frequency of severe AEs in patients treated with ceritinib was significantly higher than patients treated with crizotinib or alectinib, especially for hepatotoxicity, fatigue and some of gastrointestinal symptoms. Additionally, significant difference in the elevated lipase and amylase levels (grade ≥ 3) were detected between ceritinib and crizotinib/alectinib, whereas neutropenia was less frequent.

**Conclusions:**

ALK-TKIs were safe for ALK-positive patients. Moreover, statistically significant differences in some severe AEs among ceritinib, crizotinib and alectinib were detected in present study.

**Electronic supplementary material:**

The online version of this article (doi:10.1186/s12885-017-3405-3) contains supplementary material, which is available to authorized users.

## Background

With the discovery of anaplastic lymphoma kinase (ALK) rearrangements, small-molecule ALK tyrosine kinase inhibitors (TKIs) become the most active therapeutic areas of study in ALK-positive non-small-cell lung cancer (NSCLC) patients. Although crizotinib became recommended standard first-line therapy (https://www.nccn.org/patients/guidelines/lung-nsclc/index.html#88), acquired resistance and the development of brain metastases were the biggest obstacles during the treatment of crizotinib in ALK-positive NSCLC. Next generation ALK TKIs (ceritinib and alectinib) came into our sight and approved by the United States (US) Food and Drug Administration (FDA) for treated patients with crizotinib-intolerant, crizotinib-progressive or crizotinib-resistant ALK-positive NSCLC (http://www.accessdata.fda.gov/drugsatfda_docs/label/2015/208434s000lbl.pdf, www.accessdata.fda.gov/drugsatfda_docs/label/2014/205755lbl.pdf34s000lbl.pdf). Notably, the aforementioned three ALK-TKIs (crizotinib, ceritinib and alectinib) are by far the best available treatment options for ALK-positive NSCLC patients and show promising response rate. However, the differences of toxicity profiles among these drugs remain unclear. In clinical, toxicity profiles are usually as deciding factors for clinicians when selecting an effective regimen for ALK-positive NSCLC. Hence, it is important and necessary to choose a treatment with acceptable toxicological properties and a low influence on patients’ quality of life (QoL), especially for palliating severe treatment related symptoms. Additionally, the best treatment response with the lowest possible toxicity should be obtained in selecting patients with consideration for their complications and treatment regimen. Therefore, we performed a pooled analysis of the occurrence of severe (grade ≥ 3) toxicity according to the ALK-TKI type based on data extracted from clinical trials of ALK-positive NSCLC patients.

## Methods

### Search method

A comprehensive computerized search of the MEDLINE, EMBASE, WEB OF SCIENCE, and COCHRANE databases encompassing the period from the drugs’ inception to May 2016 was performed to identify clinical trials in English-language journals. The key words were as follows: “crizotinib,” “ceritinib,” “alectinib,” “non-small-cell lung cancer,” and “ALK-positive.” The reference lists of all pertinent studies were also manually searched. Meeting abstracts from the American Society of Clinical Oncology, the European Society for Medical Oncology and ClinicalTrials.gov were also hand-searched to identify eligible trials. Reference lists of original articles and review articles were further investigated.

According to the PICO checklist, the eligibility criteria were as follows: (1) population: patients with locally advanced or metastatic anaplastic lymphoma kinase-positive non-small-cell lung cancer; (2) intervention: crizotinib, ceritinib, or alectinib; (3) control: none; and (4) outcome: the occurrence of severe (grade ≥ 3) toxicity.

When necessary, we contacted the corresponding authors of some studies for further information. Our study was managed according to the Preferred Reporting Items for Systematic Reviews and Meta-analyses (PRISMA) guidelines (when appropriate).

### Study selection

The included studies were as follows: (i) clinical trials that researched ALK-TKIs (crizotinib, ceritinib or alectinib) in ALK-positive NSCLC patients; (ii) presented sufficient data on treatment-related adverse events (TRAEs), including grade ≥ 3 TRAEs; (iii) written in English; and (iiii) the latest article with the most complete data when multiple articles were based on the same trial. When information about AEs leading to patient withdrawal was not available, we defined all AEs as non-withdrawal toxicities if the trial described all treatment-related toxicities as “acceptable”. When information about grade ≥ 3 TRAEs was not available, we attempted to contact with the correspondence author of the study for clarification and defined the vague interpretation as “not available (NA)”. We excluded case reports, letters, commentaries, and reviews.

### Study quality assessment

Two investigators (D.S W and X.F Z) assessed the full text of non-randomized clinical trials (NRCTs) using the 9-point Newcastle-Ottawa Scale (NOS) [1]. Each study was independently evaluated by the above two investigators according to eight items. Studies were categorized into three broad perspectives, including selection, comparability and outcomes for cohort studies or exposure for case-control studies [1]. A score of 7 or greater for the studies was considered high quality. The risk of bias in the included studies was independently assessed by two investigators using the Cochrane collaboration’s tool for assessing the risk of bias in randomized control trials (RCTs) [2]. Two authors (C.L C and Z.Q Z) independently assessed each study under five main headings for the risk of bias. Differences were solved by discussion or through consulting with the senior investigator.

### Data extraction

The following data were extracted from all eligible studies: the first author’s name, publication year, number of patients evaluable for toxicity, type of ALK-TKI (crizotinib, ceritinib or alectinib), patient ethnicity, and number of patients experiencing toxicity (hepatotoxicity, neutropenia, dyspnoea, fatigue, vomiting, diarrhoea, nausea, constipation, elevated lipase and amylase levels, grade ≥ 3 and interstitial lung disease [ILD] of any grade). Ceritinib [3–7] and alectinib [8, 9] studies were excluded from the ethnicity analysis for their limited use to non-Asian patients in previous reports. Crizotinib studies were excluded from the ethnicity analysis because the data on non-Asian patients were not available, except data on the hepatotoxicity [10–19]. Ceritinib and alectinib studies were excluded from the line of treatment analysis as ceritinib and alectinib were not used for the first-line setting in present inclusive clinical trials [3–9, 20]. Studies were independently selected by two authors (H. H and Q. Z) based on the aforementioned inclusion criteria.

Safety data were collected for patients receiving crizotinib at 250 mg/day twice daily (BID), ceritinib at 750 mg/day once daily or alectinib at 600 mg BID according to the U.S. FDA-approved dose. AEs were in accordance with the criteria provided by the National Cancer Institute Common Terminology Criteria guidelines. The hepatotoxicity grade was defined in accordance with a higher value for either alanine aminotransferase or aspartate aminotransferase. Treatment-related death (TRD) and toxicities that required temporary treatment interruption were not included as withdrawal toxicities. All statistical analysis was performed with GraphPad Prism software (version 6.0; GraphPad Software, San Diego, CA). Fisher’s exact or chi-square tests were used to compare the frequencies of AEs among ALK-TKIs, if appropriate. All tests were two-tailed, and statistical significance was considered *p* < 0.05.

## Results

### Primary characteristics of selected trials

**Fig. 1:**
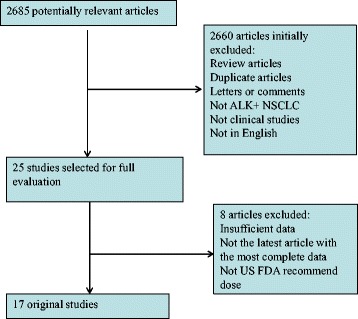
Flow diagram of the study. ALK^+^ NSCLC, anaplastic lymphoma kinase-positive non-small cell lung cancer; US, United States; FDA, Food and Drug Administration

According to our search criteria, we identified 17 trials of ALK-TKIs treated ALK-positive NSCLC patients (Fig. 1). Among 17 trials published between 2011 and 2016, 1826 ALK-positive patients were eligible for present analysis. The sample size of the eligible trials ranged from 7 to 246. The patients in 10 studies (1000 patients) received crizotinib [10–19], 5 studies (601 patients) received ceritinib [3–7], and 2 studies (225 patients) received alectinib [8, 9]. The primary characteristics of the selected studies were listed in Table 1.

**Table 1:** The primary characteristics of the selected studies

First author	Treatment	No. of patients	No. of treatment-related toxicities grade ≥ 3										
			Total	Hepatotoxicity	Neutropenia	Dyspnoea	Fatigue	Vomiting	Diarrhoea	Nausea	Constipation	Amylase increased	Elevated lipase
D.W. Kim [10]	Crizotinib	136	27	5	3	4	NA	NA	NA	NA	NA	NA	NA
Camidge, D. R. [11]	Crizotinib	149	36	6	9	1	2	1	0	1	1	0	0
Perol, M. [12]	Crizotinib	187	NA	5	NA	NA	NA	NA	NA	NA	NA	NA	NA
Shaw, Alice T. [13]	Crizotinib	172	57	27	23	7	4	2	0	2	4	NA	NA
Cao, Y. [14]	Crizotinib	40	6	4	0	0	0	2	0	0	0	0	0
Hernandez, Berta [15]	Crizotinib	10	NA	2	NA	NA	NA	NA	NA	NA	NA	NA	NA
Shaw, A. T. [3]	Ceritinib	81	46	19	NA	NA	5	5	6	6	NA	8	8
Solomon, B. J. [16]	Crizotinib	171	NA	24	19	5	5	3	4	2	3	NA	NA
Chaigneau, A. [4]	Ceritinib	10	2	1	0	0	0	0	0	0	0	0	0
Cui, S. [17]	Crizotinib	72	10	3	0	0	0	5	0	0	0	0	0
Felip, E. [5]	Ceritinib	124	NA	17	NA	NA	NA	NA	NA	NA	NA	NA	NA
Mok, T. [6]	Ceritinib	140	64	22	NA	NA	NA	6	9	8	NA	NA	NA
Cui, S. [18]	Crizotinib	56	8	2	0	0	0	5	0	0	0	0	0
Kim, D. W. [7]	Ceritinib	246	125	73	0	10	12	11	15	15	0	8	16
Ou, Sai-Hong [8]	Alectinib	138	38	2	NA	4	1	1	1	0	0	NA	NA
Shaw, A. T. [9]	Alectinib	87	35	5	1	3	0	0	0	0	0	0	0
Zhang, Q. [19]	Crizotinib	7	1	1	0	0	0	0	0	0	0	0	0

*NA* not available

### Study quality assessment and risk of bias

The methodological quality of all NRCTs (excluding the abstracts only and conferences) was summarized in Additional file 1: Table S1. The NOS results showed that the average overall score was 5.4 (range 5–7). No major flaws of the included RCTs were detected in assessing their risk of bias. However, the expected absence of blinded intervention was a common caveat. We summarized the detailed assessment of the risk of bias in Additional file 2: Table S2.

### Frequency of treatment-related death according to the ALK-TKI type

**Fig. 2:**
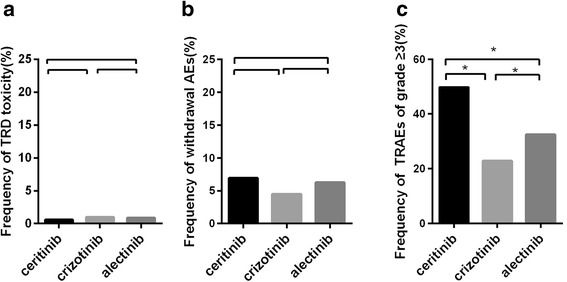
Frequency of grade ≥ 3 AEs, including TRD (**a**), withdrawal toxicities (**b**) and overall frequency (**c**), according to ALK-TKIs type. TRD, treatment-related death and AEs, adverse events. Asterisks indicate statistically significant differences

Treatment-related death (TRD) was reported in 12 of the 1365 evaluable patients, resulting in an overall prevalence of 0.9%. The main cause of such death was ILD or pneumonitis (5 of 12 patients). Eight of 803 patients (1.0%) experienced TRD (due to ventricular arrhythmia and an unknown cause in one patient each, pulmonary embolism in 2 cases and ILD or pneumonitis in 4 cases) in the crizotinib group, 2 of 337 patients (0.6%) experienced TRD (due to ILD and multi-organ failure in one patient each) in the ceritinib group and 2 of 225 patients (0.9%) experienced TRD (due to intestinal perforation and haemorrhage in one patient each) in the alectinib group. However, the significantly difference of TRD among the three cohorts (crizotinib vs. ceritinib, *P* = 0.732; crizotinib vs. alectinib, *P =* 1.000; ceritinib vs. alectinib, *P* = 0.683) were not detected (Fig. 2a).

### Identification of withdrawal toxicities according to the ALK-TKI type

Subset of NSCLC patients with ALK-positive status terminated the ALK-TKI treatment for adverse events. The overall frequency of AEs resulted in treatment withdrawal was 5.5% (85 of 1543 evaluable patients). Whereas, the significant difference of withdrawal AEs were not observed in the ceritinib group and crizotinib group (6.9% vs. 4.5%, *P* = 0.064), alectinib and crizotinib group (5.8% vs. 4.5%, *P =* 0.432), or ceritinib and alectinib group (*P =* 0.563) (Fig. 2b).

### Frequency of grade ≥ 3 TRAEs according to the ALK-TKI type

The overall frequency of TRAEs (grade ≥ 3) was significant greater in patients treated with ceritinib than those treated with crizotinib (49.7% vs. 22.9%; OR 3.32, 95% CI 2.56–4.29, *P <* 0.001). Same result was detected between patients treated with ceritinib and those treated with alectinib (49.7% vs. 32.4%, OR 2.06, 95% CI 1.48–2.87, *P <* 0.001). However, the overall frequency of grade ≥ 3 TRAEs was significantly lower in the crizotinib cohort than alectinib (22.9% vs. 32.4%, OR 0.62, 95% CI 0.44–0.87, *P =* 0.049) (Fig. 2c).

### Frequency of severe TRAEs (grade ≥ 3) between different ALK-TKI types

**Fig. 3:**
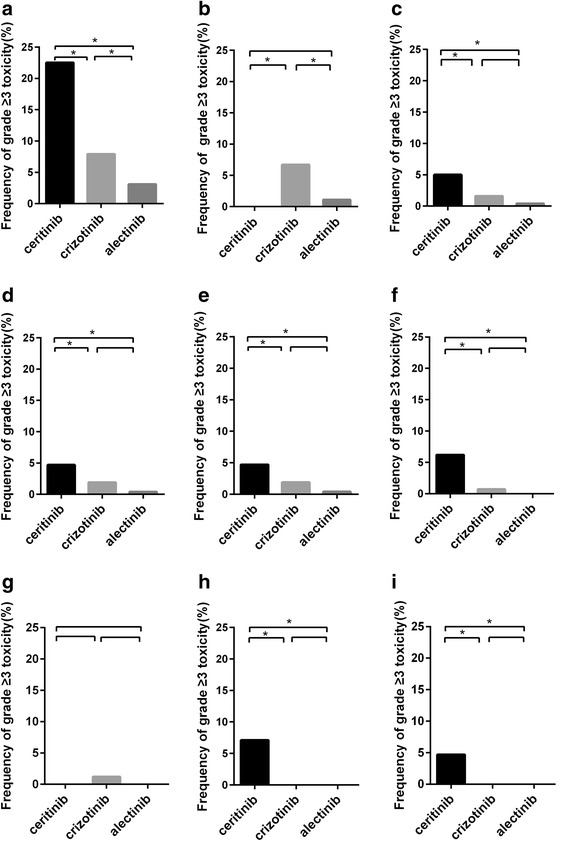
Frequency of AEs grade ≥ 3, including hepatotoxicity (**a**), neutropenia (**b**), fatigue (**c**), vomiting (**d**), diarrhoea (**e**), nausea (**f**), constipation (**g**), elevated lipase level (**h**) and elevated amylase level (**i**) according to the ALK-TKIs type. AEs, adverse events. *Asterisks* indicate statistically significant differences

The frequency of severe hepatotoxicity was significantly greater in patients treated with ceritinib than patients treated with crizotinib (22.5% vs. 7.9%, OR 3.38, 95% CI 2.50–4.56, *P* < 0.001) and patients treated with crizotinib compared with those treated with alectinib (7.9% vs. 3.1%, OR 2.67, 95% CI 1.22–5.87, *P* = 0.011). Meanwhile, patients in the ceritinib group experienced significantly higher frequency of severe hepatotoxicity than patients in alectinib group (22.5% vs. 3.1%, OR 9.02, 95% CI 4.15–19.60, *P <* 0.001) (Fig. 3a).

Neutropenia of grade ≥ 3 was significantly less frequent for ceritinib than crizotinib (0.0% vs. 6.7%, OR 0.03, 95% CI 0.01–0.44, *P* < 0.001). However, neutropenia of grade ≥ 3 was significantly more frequent for crizotinib compared with alectinib (6.7%% vs. 1.1%, OR 6.20, 95% CI 0.85–45.41, *P* = 0.040). Significant difference was not observed between ceritinib and alectinib cohort (0.0% vs. 1.1%, OR 0.11, 95% CI 0.01–2.82, *P* = 0.254) (Fig. 3b).

The frequency of grade ≥ 3 dyspnoea did not differ significantly between ceritinib and crizotinib (3.9% vs. 2.1%, OR 1.88, 95% CI 0.85–4.16, *P* = 0.114), crizotinib and alectinib (2.1% vs. 3.1%, OR 0.67, 95% CI 0.28–1.65, *P* = 0.383), as well as ceritinib and alectinib (3.9% vs. 3.1%, OR 1.27, 95% CI 0.48–3.38, *P* = 0.638).

The frequency of fatigue (grade ≥ 3) was significantly higher for ceritinib than crizotinib (5.0% vs. 1.6%, OR 3.17, 95% CI 1.47–6.85, *P* = 0.002) and alectinib (5.0% vs. 0.4%, OR 11.90, 95% CI 1.57–90.11, *P* = 0.002). Nonetheless, there was no significant difference between crizotinib and alectinib (1.6% vs. 0.4%, OR 3.76, 95% CI 0.48–29.27, *P* = 0.175) (Fig. 3c).

Vomiting of grade ≥ 3 was significantly more frequent for ceritinib compared with crizotinib (4.6% vs. 1.9%, OR 2.43, 95% CI 1.21–4.87, *P* = 0.010) and alectinib (4.6% vs. 0.4%, OR 10.80, 95% CI 1.45–80.90, *P* = 0.004). Although patients with crizotinib showed a higher trend of vomiting of grade ≥ 3 than alectinib, statistically difference was not detected (1.9% vs. 0.4%, OR 5.37, 95% CI 0.70–41.25, *P* = 0.080) (Fig. 3d).

Similarly, the frequency of diarrhoea (grade ≥ 3) was significantly greater in patients treated with ceritinib than those treated with crizotinib (6.3% vs. 0.6%, OR 11.12, 95% CI 3.89–31.80, *P* < 0.001) and alectinib (6.3% vs. 0.4%, OR 15.03, 95% CI 2.04–111.00, *P* < 0.001), whereas it was not significantly higher in patients treated with crizotinib than those with alectinib (0.6% vs. 0.4%, OR 1.63, 95% CI 0.18–14.65, *P* = 1.000) (Fig. 3e).

A similar pattern was observed in the frequency of grade ≥ 3 nausea. Nausea of grade ≥ 3 also occurred significantly more often in ceritinib cohort than crizotinib (6.1% vs. 0.7%, OR 8.57, 95% CI 3.29–22.30, *P* < 0.001) and alectinib cohort (6.1% vs. 0.0%, OR 29.66, 95% CI 1.80–488.00, *P* < 0.001). Statistical significance was not yet reached between the crizotinib and alectinib cohorts (0.7% vs. 0.0%, OR 4.49, 95% CI 0.25–81.56, *P =* 0.329) (Fig. 3f).

The frequency of constipation grade ≥ 3 was low for all three ALK-TKIs (1.2% for crizotinib and 0.0% for ceritinib or alectinib) and did not differ significantly among the above three cohorts (crizotinib vs. ceritinib, OR 6.61, 95% CI 0.38–115.10, *P =* 0.115; ceritinib vs. alectinib, OR 1.05, 95% CI 0.02–53.40, *P =* 1.000) (Fig. 3g).

Elevated lipase level of grade ≥ 3 was significantly more frequent for ceritinib than crizotinib (7.1% vs. 0.0%, OR 50.72, 95% CI 3.07–838.20, *P* < 0.001) and alectinib (7.1% vs. 0.0%, OR 13.68, 95% CI 0.82–227.30, *P* = 0.010). Among patients received crizotinib, significant difference was not observed when compared with alectinib (*P* = 1.000) (Fig. 3h). Likewise, the frequency of elevated amylase level of grade ≥ 3 was significantly higher in patients treated with ceritinib than those treated with crizotinib (4.7% vs. 0.0%, OR 33.31, 95% CI 1.99–557.90, *P* < 0.001) and alectinib (4.7% vs. 0.0%, OR 8.98, 95% CI 0.53–151.00, *P* = 0.038). Statistical significance was not observed between crizotinib and alectinib (*P* = 1.000) (Fig. 3i).

Moreover, the frequency of peripheral oedema (grade ≥ 3) was low for all three ALK-TKIs (0.1% for crizotinib, 0.0% for ceritinib and 0.4% for alectinib) and did not differ significantly among the three cohorts (data not shown). A similar pattern was observed for the frequency of ILD of any grade. The frequency of ILD of any grade was low for all three ALK-TKIs (1.3% for crizotinib, 0.8% for ceritinib and 0.0% for alectinib) and did not differ significantly among the three cohorts (data not shown).

To investigate whether the line of treatment might affect the incidence of toxicities (grade ≥ 3), we analysed the frequency of such toxicities in patients receiving crizotinib between the first-line and second-line setting, and no statistical significance was detected (Additional file 3: Table S3).

### Frequency of TRAEs grade ≥ 3 according to the patient ethnicity

In this study, no statistically significant difference of severe crizotinib-related hepatotoxicity (grade ≥ 3) was reported between Asians and non-Asians (5.7% vs. 3.6%, OR 1.65, 95% CI 0.61–4.42, *P* = 0.333).

## Discussion

Lung cancer is the leading cause of cancer death because most patients are diagnosed at an advanced stage [21]. Alleviating and preserving patients’ QoL are important treatment aims. Targeted therapy is one of the major modalities of medical treatment for cancer with minor side effects and good efficacy. As shown in our study, the frequency of TRD in the ceritinib, crizotinib, and alectinib groups was no more than 1% (0.6%, 1.0%, and 0.9%, respectively). The frequencies of AEs leading to treatment withdrawal in the ceritinib, crizotinib, and alectinib groups were 6.9%, 4.5%, and 5.8%, respectively. Our pooled analysis show minor side effects of ALK TKIs for treated patients with ALK positive NSCLC.

Present study further systematically analyzed the differences of treatment related severe AEs among ALK-TKIs. For ALK-TKIs, the majority of AEs may be related to inhibiting the specific molecular target in normal tissues. In present study, our pooled analysis revealed that the frequencies of grade ≥ 3 hepatotoxicity induced by ceritinib, crizotinib or alectinib were 22.5%, 7.9% or 3.3%, respectively. These results indicated that long-term exposure to ceritinib was associated with an increased frequency of hepatotoxicity compared to crizotinib or alectinib. A previous study showed crizotinib was an oral, ATP-competitive, selective inhibitor of the ALK, mesenchymal-epithelial transition factor (MET)/hepatocyte growth factor (HGF) receptor tyrosine kinases [22]. Compared with crizotinib, ceritinib does not inhibit the kinase activity of MET; however, it inhibits insulin-like growth factor 1 (IGF-1) and insulin receptor, which mediate growth and development [3]. IGF receptor was ubiquitous at the cell surface and existed on the surface of cells [23]. As a result, patients who were treated with ceritinib have a typical hepatotoxicity. Meanwhile, alectinib is a novel, highly selective oral ALK inhibitor [24]. As a result, patients treated with alectinib present with the lowest hepatotoxicity among these drugs. Moreover, differences in the severe AEs between first-line and second-line crizotinib were not detected. Similarly, the overall safety profile of first-line ceritinib in advanced ALK-rearranged NSCLC (NCT01828099) was consistent with those safety profiles of ceritinib in ALK-rearranged NSCLC patients who had progressed on multiple lines of chemotherapy (NCT01283516 and NCT01685138) [3, 5, 25]. However, first-line alectinib study (NCT02075840) is still ongoing globally [26]. Given the possible long-term exposure of ALK-positive patients to ALK-TKIs, it is important to adequately manage hepatotoxicity while balancing the QoL and treatment compliance. The general risk factors, including older age; female sex; HIV, HBV, or HCV infection; pregnancy; excess alcohol intake; smoking and genetic variability; and, especially, exacerbation of pre-existing liver disease could not be excluded as a possible mechanism for hepatotoxicity [27]. For patients experiencing hepatotoxicity grade ≥ 3, discontinuation generally reverses hepatotoxicity. Patients with the aforementioned general risk factors require more attention. Other feasible options include temporarily suspending the agent, reducing the dose, permanently discontinuing the medication or changing to another ALK-TKI.

In the meantime, our pooled analysis showed that neutropenia grade ≥ 3 occurs significantly more often among patients treated with crizotinib than those treated with ceritinib or alectinib. As mentioned earlier, crizotinib is a multi-target receptor TKI of both ALK and c-Met/hepatocyte growth factor (HGF) receptor kinases. HGF, which was reportedly produced by bone marrow stromal cells, promotes haematopoiesis via the c-Met receptor [28]. Additionally, a previous study indicated that the pathogenesis of neutropenia might be associated with inhibitory action against the c-Met receptor [29]. Therefore, we inferred the inhibitory action of crizotinib against the c-Met receptor maybe the source of the difference. Another study proposed that an idiosyncratic drug-induced neutropenia was mediated by the immune response [30]. The immune response might be another source of the differences. The pathogenesis and mechanism of crizotinib-induced neutropenia remained controversial. The dose reduction of crizotinib and subsequent administration of other ALK-TKIs might be a feasible method.

Fatigue grade ≥ 3 occurs significantly more frequent among patients treated with ceritinib than those treated with crizotinib or alectinib. As previously mentioned, ceritinib inhibited the IGF-1 and insulin receptors. In a previous study, increasing the IGF-1 level resulted in improved well-being and lowering the IGF-1 levels led to more complaints of fatigue [31]. Moreover, IGF-1 receptors were expressed in skeletal muscle, and abnormal skeletal muscle metabolism was associated with peripheral fatigue [32]. Hence, we deduced that the pathogenesis of fatigue might be associated with the inhibitory action of ceritinib against the IGF-1 receptor. Dose reduction, subsequent administration of other ALK-TKIs, or supplementation of growth hormone or/and IGF-1 might be effective approaches.

In our pooled analysis, the frequency of vomiting, diarrhoea or nausea grade ≥ 3 was significantly higher in patients treated with ceritinib than those treated with crizotinib or alectinib. However, the frequency of constipation grade ≥ 3 did not significantly differ among the three cohorts. We speculated that these differences in the frequency of vomiting, diarrhoea and nausea might be related to the oral formulation, meal type and liver function disorders [33]. An effective and promising approach for maximizing patient drug exposure when patients start a new therapy involves preparing patients and physicians to expect adverse effects; dispensing the supportive treatment before the patient leaves the office and takes the first dose; and implementing proactive methods upfront as part of a treatment package [34].

Furthermore, the frequency of elevated lipase and amylase level grade ≥ 3 in the ceritinib cohort occurs significantly more often than in the crizotinib and alectinib cohorts. These differences in the frequency of elevated lipase and amylase levels might be related to the mechanism of inhibiting a specific molecular target. Previous studies demonstrated that high-affinity IGF-I receptors are expressed on pancreatic alpha and beta cells and in acinar tissue [35]. As we know, only ceritinib among these ALK-TKIs inhibited IGF-1 and insulin receptors. Hence, the irreversible tyrosine kinase blockade mediated by ceritinib might result in higher elevated lipase and amylase levels compared with crizotinib or alectinib. Additionally, the overall frequency of TRAEs grade ≥ 3 in the ceritinib cohort was significantly higher than in the crizotinib or alectinib cohort. As shown in our study, patients with ceritinib generally experienced a higher trend of severe AEs than those treated with crizotinib or alectinib. Therefore, ALK inhibition with crizotinib or alectinib might be superior to ceritinib in most situations for treating ALK-positive NSCLC patients and have manageable toxicities. Moreover, the frequency of severe AEs among different ALK-TKI types could aid clinicians in choosing the most suitable treatments for ALK-positive NSCLC patients to alleviate the risk of some toxicity types.

With respect to ceritinib, a higher rate of grade ≥ 3 hepatotoxicity and gastrointestinal symptoms was the first burden compared with crizotinib and alectinib.

Although the recommended daily ceritinib dose of 750 mg taken under fasting conditions, a report has shown ceritinib administered at non-fasted state that suggest decreased toxicity in gastrointestinal symptoms, such as nausea [36]. Notably, ceritinib at a dose of 750 mg administered under non-fasting conditions is expected to result in increased systemic exposure and may increase exposure-dependent adverse drug reactions, which is associated with a higher frequency of grade ≥ 3 hepatotoxicity [37]. To determine the suitable dose with food to improve gastrointestinal tolerability, a randomized trial is currently being conducted to estimate whether lower doses (450 mg or 600 mg) of ceritinib at non-fasted state provides similar steady-state systemic exposure compared to the 750 mg dose taken under fasting conditions in patients with ALK-positive NSCLC (https://www.clinicaltrials.gov/ct2/show/NCT02299505?term%C2%BCLDK378&rank%C2%BC22). It is important and feasible to select patients for a treatment according to their complications and general risk factors before starting TRAEs, especially for patients with long-term exposure to ALK-TKIs [33]. Based on present pooled analysis of large datasets, the clinicians can choose more suitable treatment for ALK-positive NSCLC patients in terms of safety and side effects.

The present study had several limitations. First, this study systematically analysed all published clinical trials and detected statistically significant of some severe AEs among ceritinib, crizotinib and alectinib. However, further prospective RCTs with a high sample size are required. Second, although we contacted the corresponding authors of some studies for further information, when necessary, some information was not obtained. Third, studies that were not in English and unpublished studies were not included.

## Conclusion

Our study further demonstrated that ALK-TKIs are safe for ALK-positive NSCLC patients. Moreover, the statistically significant differences of some severe AEs among ceritinib, crizotinib and alectinib were detected.

## Additional files

Additional file 1: Table S1. Nine-point Newcastle Ottawa scale scores for the non-randomized controlled trials (DOC 39 kb)

Additional file 2: Table S2. Risk of bias in randomized controlled trials (DOC 31 kb)

Additional file 3: Table S3. Frequency of crizotinib-related AEs grade ≥ 3 according to the line of treatment (DOC 40 kb)

## Abbreviations

AEs: Adverse events
ALK: Anaplastic lymphoma kinase
CI: Confidence interval
FDA: Food and Drug Administration
HGF: Hepatocyte growth factor
IGF-1: Insulin-like growth factor 1
ILD: Interstitial lung disease
MET: Mesenchymal epithelial transition factor
NOS: Newcastle-Ottawa Scale
NRCTs: Non-randomized clinical trials
NSCLC: Non-small cell lung cancer
OR: Odds ratio
QoL: Quality of life
RCTs: Randomized clinical trials
TKIs: Tyrosine kinase inhibitors
TRAEs: Treatment-related adverse events
TRD: Treatment-related death
