# Supplementary material for: Pooled safety analyses of ALK-TKI inhibitor in ALK-positive NSCLC
Source: BMC Cancer. 2017 Jun 12;17:412. doi: 10.1186/s12885-017-3405-3 (PMC5469041; doi:10.1186/s12885-017-3405-3)
Supplement: Supplementary file 1 — Nine-point Newcastle Ottawa scale scores for the non-randomized controlled trials (DOC 39 kb) [file 12885_2017_3405_MOESM1_ESM.doc]

| **Table S1** Nine-point Newcastle Ottawa scale scores for the non-randomized controlled trials | | | | | | | | |
| --- | --- | --- | --- | --- | --- | --- | --- | --- |
|  | Selection  (Maximum of four stars) | | | | Comparability (Maximum of two stars) | Outcome  (Maximum of three stars) | | |
|  | Representativeness of the exposed cohort | Selection of the non-exposed cohort | Ascertainment of exposure | Demonstration the outcome of interest was not present at the start of the study | Comparability of cohorts on the basis of the design or analysis | Assessment of the outcome | Was follow-up long enough for the outcome to occur | Adequacy of follow-up of cohorts |
| Camidge D [14] |  |  | * | * |  | * | * | * |
| Cao, Y.[17] |  |  | * | * |  | * | * | * |
| Shaw, A. T.[6] |  |  | * | * |  | * | * | * |
| Cui, S.[20] |  |  | * | * |  | * | * | * |
| Cui, S.[21] |  |  | * | * | ** | * | * | * |
| Kim, D. W.[10] |  |  | * | * |  | * | * | * |
| Ou, Sai-Hong [11] |  |  | * | * |  | * | * | * |
| Shaw, A.T.[12] |  |  | * | * |  | * | * | * |
| Zhang, Q.[22] |  |  | * | * | ** | * | * | * |
