# Supplementary material for: Pooled safety analyses of ALK-TKI inhibitor in ALK-positive NSCLC
Source: BMC Cancer. 2017 Jun 12;17:412. doi: 10.1186/s12885-017-3405-3 (PMC5469041; doi:10.1186/s12885-017-3405-3)
Supplement: Supplementary file 2 — Risk of bias in randomized controlled trials (DOC 31 kb) [file 12885_2017_3405_MOESM2_ESM.doc]

| **Table S2** Risk of bias in randomized controlled trials | | | | | | |
| --- | --- | --- | --- | --- | --- | --- |
| Study | Selection bias | Performance bias | Detection bias | Attrition bias | Reporting bias | Other bias |
| Shaw, Alice T.[16] | Unclear – no description of random sequence generation or allocation concealment | High – no blinding of participants or personnel | High – no blinding of participants or personnel | Low – all trial participants were accounted for | Low – no selective reporting | Low |
| Benjamin J. Solomon [19] | Unclear – no description of random sequence generation or allocation concealment | High – no blinding of participants or personnel | High – no blinding of participants or personnel | Low – all trial participants were accounted for | Low – no selective reporting | Low |
