# Supplementary material for: Pooled safety analyses of ALK-TKI inhibitor in ALK-positive NSCLC
Source: BMC Cancer. 2017 Jun 12;17:412. doi: 10.1186/s12885-017-3405-3 (PMC5469041; doi:10.1186/s12885-017-3405-3)
Supplement: Supplementary file 3 — Frequency of crizotinib-related AEs grade ≥ 3 according to the line of treatment (DOC 40 kb) [file 12885_2017_3405_MOESM3_ESM.doc]

| **Table S3** Frequency of crizotinib-related AEs grade ≥3 according to the line of treatment | | | | |
| --- | --- | --- | --- | --- |
| AE | Frequency of grade ≥3 toxicity (%) | |  | Fisher’s exact test P value (OR, 95% CI) |
|  | First-line | Second-line |  | First-line vs. Second-line |
| Hepatotoxicity | 14.0 | 15.7 |  | 0.664(0.88, 0.49-1.58) |
| Neutropenia | 10.7 | 13.4 |  | 0.438(0.77, 0.41-1.48) |
| Dyspnoea | 2.8 | 4.1 |  | 0.517 (0.68, 0.21–2.19) |
| Fatigue | 2.8 | 2.3 |  | 0.775 (1.21, 0.32–4.60) |
| Vomiting | 1.7 | 1.2 |  | 0.680 (1.46, 0.24–8.83) |
| Diarrhoea | 2.3 | 0.0 |  | 0.123 (8.89, 0.48–166.60) |
| Nausea | 1.1 | 1.2 |  | 1.000 (0.97, 0.13–6.94) |
| Constipation | 1.7 | 2.3 |  | 0.720 (0.72, 0.16–3.27) |
| Peripheral oedema | 0.6 | 0.0 |  | 1.000(2.92, 0.12-72.12) |
| Amylase increased | NA | NA |  | NA |
| Elevated lipase level | NA | NA |  | NA |

NA, not available.
